# Medial pulvinar stereoelectroencephalographic biomarkers associated with deep brain stimulation response in focal drug‐resistant epilepsy

**DOI:** 10.1111/epi.70046

**Published:** 2025-12-04

**Authors:** Ionuț‐Flavius Bratu, Romain Carron, Audrey Clement, Samuel Medina Villalon, Fabrice Bartolomei, Francesca Pizzo

**Affiliations:** ^1^ Department of Epileptology and Cerebral Rhythmology Timone Hospital Marseille France; ^2^ Systems Neuroscience Institute, French National Institute of Health and Medical Research (UMR 1106) Aix‐Marseille University Marseille France; ^3^ Medico‐Surgical Unit of Epileptology, Functional and Stereotactic Neurosurgery Timone Hospital Marseille France

**Keywords:** DBS, permutation entropy, pulvinar, SEEG

## Abstract

Thalamic deep brain stimulation (DBS) represents an emerging therapeutic option for patients with focal drug‐resistant epilepsy who are ineligible for or have failed resective surgery. To optimize outcomes and guide DBS lead placement, thalamic stereoelectroencephalography (SEEG) has been proposed. This monocentric retrospective study aimed to identify interictal and ictal SEEG biomarkers of the medial pulvinar (PuM) associated with favorable PuM‐DBS response. Six patients (four female, two male) underwent SEEG including PuM sampling, were deemed unsuitable for resective surgery, and subsequently received bilateral PuM‐DBS. In total, eight PuMs were sampled in SEEG: four bilaterally (two patients) and four unilaterally (four patients). The SEEG exploration covered both the PuM and the ipsilateral epileptogenic zone network (EZN) in four patients, whereas in the other two the EZN was bilateral but PuM sampling was unilateral. All SEEG signal analyses were performed on the PuM sampling contacts available in each patient. Interictal SEEG analysis included spike rates and nonlinear functional connectivity (h2), whereas ictal analyses combined visual inspection with quantitative biomarkers: epileptogenicity index (EI), connectivity epileptogenicity index (cEI), permutation entropy index (PEI), and delta entropy (ΔE). Two patients were responders (≥50% seizure reduction at 1 year). PuM spike rates, connectivity, and EI and cEI values did not differentiate responders from nonresponders. In contrast, entropy‐based measures were significantly higher in responders: PEI (false discovery rate [FDR]‐*p* = .024) and ΔE (FDR‐*p* = .034). These findings suggest that ictal PuM complexity disruption, quantified through entropy‐based SEEG metrics (PEI and ΔE), may represent a candidate biomarker of response to medial pulvinar DBS and warrants validation in larger cohorts.

**Key points**

- This study examined medial pulvinar SEEG biomarkers associated with deep brain stimulation response.
- Visual SEEG analysis, spike rates, and the (connectivity) epileptogenicity indices did not distinguish responders from nonresponders.
- Medial pulvinar ictal entropy‐based SEEG measures (PEI and ΔE) were higher in the responder group.
- PEI represents the ratio between the maximum postictal entropy and the minimum ictal entropy.
- ΔE represents the difference between the baseline mean entropy and the minimum ictal entropy.

## 1 INTRODUCTION

Thalamic deep brain stimulation (DBS) is increasingly used in patients with focal drug‐resistant epilepsy (fDRE) who are ineligible for or have failed resective surgery.^1^ To optimize outcomes, stereoelectroencephalographic (SEEG) exploration of multiple thalamic nuclei has been proposed to guide DBS lead placement.^2^ It has been hypothesized that the optimal thalamic DBS target is the nucleus most engaged during seizure propagation. This hypothesis, however, remains to be explored further.^3^ Because most studies^4^, ^5^ predominantly sample only one thalamic nucleus at a time, the relative involvement of specific nuclei cannot be reliably assessed, and large multisite thalamic recording datasets remain scarce. Current literature^2^, ^6^, ^7^ shows that when multiple thalamic sites are sampled simultaneously, a given nucleus tends to be recruited with a consistent electrophysiological profile across seizures that share the same cortical onset signature, whereas different seizure types or onset patterns within the same patient may engage the thalamus differently. Complementarily, Damiani et al.^6^ showed that the clinical efficacy of thalamic stimulation depends on hodological matching—that is, alignment between the stimulated thalamic nucleus and the seizure onset zone connectivity pattern—rather than on the mere presence of ictal activation. Medial pulvinar (PuM) DBS appears to reduce seizure frequency and severity and to improve cognition in selected fDRE patients.^8^, ^9^ However, informed thalamic DBS targeting for optimal outcomes lacks clear electrophysiological criteria.

This monocentric retrospective study aimed to identify interictal and ictal PuM SEEG features associated with favorable PuM‐DBS response.

## 2 MATERIALS AND METHODS

### 2.1 Patient selection

We included fDRE patients managed at the Timone Hospital Epileptology Department (2000–2025) who underwent SEEG (including PuM sampling), were deemed unsuitable for resective epilepsy surgery after invasive exploration, and received bilateral PuM‐DBS, with a minimum follow‐up of 1 year (detailed inclusion/exclusion criteria are given in Pizzo et al.).^9^ Informed written consent was obtained from all patients, and the study was approved by the local ethics committee (Assistance Publique–Hôpitaux de Marseille, PADS25‐187).

### 2.2 SEEG method and signal processing

Noninvasive electrical–clinical hypothesis‐driven SEEG explorations were performed using 8–18‐contact electrodes (Dixi Medical or Alcis, 2‐mm contact length, 1.5‐mm spacing, .8‐mm diameter). By local standard departmental practice, beyond the assessment of epileptogenic networks, the H (or H′) electrode is also used for functional mapping, particularly for language. Accordingly, bilateral implantation is performed when required by the electroclinical hypothesis and the need for language lateralization assessment. This electrode samples, from lateral to mesial, the lateral posterior temporal cortex/planum temporale, Heschl's gyrus, and the pulvinar. SEEG recordings were acquired on 128‐ or 256‐channel Natus systems at sampling rates of 256, 512, 1024, or 2048 Hz. Hardware filters included a high‐pass filter (.16 Hz cutoff at −3 dB) and an antialiasing low‐pass filter with cutoff of 97, 170, 340, or 680 Hz, depending on the acquisition rate.

All SEEG signal analyses were performed on nonartifacted recordings in bipolar montage using AnyWave software (https://meg.univ‐amu.fr/AnyWave/download.html).^10^ For functional connectivity assessment, one gray matter contact pair (showing the highest signal amplitude) was selected per region.

### 2.3 Interictal analysis

Six 10‐min SEEG segments per patient were analyzed: three from wakefulness and three from non‐rapid eye movement sleep, selected ≥2 h postseizure and distributed across different times of day and night, over the 2 weeks of video‐SEEG exploration.^11^ Spike rates were computed using the Delphos AnyWave plug‐in (https://meg.univ‐amu.fr/doku.php?id=plugins:delphos).^12^ Functional connectivity was analyzed using pairwise nonlinear regression based on the h2 coefficient, which quantifies the amplitude correlation between two signals using piecewise linear regression.^13^ h2 computations were performed employing AnyWave using a 4‐s sliding window, 1‐s step, and maximum lag of .1 s. Analyses were conducted on broadband (.5–80 Hz) signals and separately within canonical electroencephalographic subbands: delta (.5–3.4 Hz), theta (3.4–7.4 Hz), alpha (7.4–12.4 Hz), beta (12.4–24 Hz), and gamma (24–80 Hz).^11^ Node strength values were calculated in MATLAB (v2023b, Windows). Pairwise h2 values yielded a connectivity matrix representing interdependence between network nodes. Mean node strength was calculated as the average of all h2 values involving a given node, computed across sliding windows and normalized by the number of connected nodes to enable cross‐subject comparison.^14^ PuM mean node strength was assessed relative to all other implanted regions, as well as specifically to ipsilateral or contralateral regions or specific networks (epileptogenic zone network [EZN], propagation zone network [PZN], noninvolved zone networks [NIZ]).

### 2.4 Ictal analysis

Three spontaneous seizures per patient were analyzed to capture variability in EZN dynamics, with respect to the seizure type and seizure onset pattern (details are provided in Supplementary Materials–Explanatory Tables, Table S1). For each seizure, the epileptogenicity index (EI),^15^ connectivity epileptogenicity index (cEI),^16^ permutation epileptogenicity index (PEI),^17^ and delta entropy (ΔE)^18^ were computed using dedicated AnyWave plug‐ins (https://meg.univ‐amu.fr/doku.php?id=plugins:ei). EI quantifies the relative contribution of each structure to seizure generation by combining the energy ratio between high‐frequency (β–γ) and low‐frequency components with the timing of this spectral shift relative to the earliest recruited region. Physiologically, a higher EI reflects a structure that both activates earlier and shows a stronger increase in high‐frequency activity, thus exhibiting greater intrinsic epileptogenicity. cEI extends this measure by integrating directed functional connectivity (“out‐degree”), thereby accounting for each region's influence on the rest of the network. Biologically, cEI identifies structures that not only generate but also drive ictal synchronization, acting as network leaders within the epileptogenic system. Entropy‐based metrics complement these indices by quantifying signal complexity. PEI measures the ratio between maximum postictal and minimum ictal entropy, indicating the postictal rebound or overshoot of complexity, whereas ΔE represents the difference between baseline mean and minimum ictal entropy, indexing the depth of ictal simplification. Low ictal entropy corresponds to highly synchronized, regular activity, whereas elevated postictal entropy reflects a return—or overshoot—toward desynchronized, noiselike dynamics characteristic of epileptogenic networks.

Biomarker bipolar contact channel values were first normalized by the maximum at segment (spike rates) or seizure (ictal biomarkers) level. The highest normalized value across all segments (sleep, wakefulness, or both) or seizures was then extracted. Brain region spike rateₘₐₓ, EIₘₐₓ, cEIₘₐₓ, PEIₘₐₓ, or ΔEₘₐₓ was defined as the highest of the contact‐level maxima among all contacts sampling that region.^17^, ^19^, ^20^ Each bipolar SEEG contact channel was classified as EZN, PZN, or NIZ based on clinical analysis incorporating EI, PEI, and cEI methods.^17^ The prevailing paradigm underlying the SEEG method posits that epileptogenic processes are best described as hierarchy organized networks: EZN, PZN, and NIZ.^21^ These subnetworks differ in their electrophysiological, biological, and connectivity features, as well as in how and when they become engaged during seizures. The EZN has the highest epileptogenicity and comprises the brain regions representing the seat of seizure generation and showing early, most of the time faster activities and more intense electrophysiological changes. The PZN includes fewer epileptogenic regions that exhibit less altered biological properties and under normal conditions would not be expected to initiate seizures. Recruitment into the PZN is typically delayed, and the activity tends to present at slower frequencies. The NIZ include regions that remain electrophysiologically salient throughout the ictal period. Distinguishing among these networks is a fundamental task in interpreting SEEG, especially using quantitative biomarkers, and carries meaningful implications for prognosis and therapeutic targeting.^22^ Using validated thresholds,^17^, ^20^ contacts with EI ≥ .4 and/or cEI ≥ .65 and/or PEI ≥ .72 were defined as EZN; those with .1 < EI < .4 and/or .3 < cEI < .65 and sustained ictal discharge were classified as PZN; all others were considered NIZ. Each brain area was subsequently labeled as EZN, PZN, or NIZ based on the most epileptogenic bipolar contact sampling that region.

### 2.5 Regions of interest

Pre‐SEEG cerebral T1‐weighted magnetic resonance imaging (MRI) scans were employed for anatomical segmentation (volumetric segmentation and cortical surface reconstruction obtained using the recon‐all pipeline of FreeSurfer, http://surfer.nmr.mgh.harvard.edu)^23^ and Virtual Epileptic Patient atlas‐based parcellation (https://ins‐amu.fr/vep‐atlas).^24^ Thalamic nuclei were segmented using an in‐house pipeline based on the 7TAMIbrain workflow.^25^ Brain extraction was performed with HD‐BET^26^ and nonlinear registration to the 7TAMIbrain template was achieved using ANTs SyN.^27^ The 7TAMIbrain atlas provides high‐resolution segmentation of basal ganglia and thalamic nuclei, including the pulvinar, with submillimetric registration accuracy validated against manual expert delineations on 7‐T MRI. For the pulvinar, comparison with the adapted electronic Morel atlas yielded Dice = .76 ± .05, volumetric similarity = .96 ± .01, and balanced average Hausdorff distance = .26 ± .07 mm, demonstrating good correspondence. Thalamic and basal ganglia parcellations were then projected and merged into the Virtual Epileptic Patient (VEP) atlas in each patient's MRI space.^28^ Coregistration of the pre‐SEEG MRI and post‐SEEG CT scans was performed using GARDEL software (https://meg.univ‐amu.fr/doku.php?id=epitools:gardel),^29^ enabling semiautomated segmentation and localization of SEEG contacts within the VEP–7TAMIbrain anatomical framework, including thalamic nuclei.

### 2.6 DBS and follow‐up

Pulvinar DBS was applied bilaterally in all patients included in this study following SEEG exploration. DBS four‐contact leads (Medtronic 3389 or SenSight B33005, with extensions 37086 or B34000) were implanted at the site of the most distal PuM‐sampling SEEG contact, using ROSA robotic assistance (Medtech, Zimmer Biomet). All patients were implanted with a Medtronic Percept PC system delivering constant‐current stimulation. Bipolar stimulation (between the two best located contacts) was initiated at 145 Hz, with a 90‐μs pulse width, in cyclic mode (5 min ON/30 s OFF), and amplitude generally starting at 1 mA. In constant‐current mode, the device automatically adjusted the delivered voltage to account for impedance variations, whereas the current amplitude was titrated during follow‐up according to clinical tolerance and responsiveness. During stimulation titration, the patients reported the following side effects: P3, left upper limb and leg dysesthesia; P4, facial and right arm and leg paresthesia; P5, bilateral hand paresthesia; and P6, bilateral upper limb paresthesia (details are provided in Supplementary Materials–Explanatory Tables, Table S2). Patients were followed every 3 months with neurological–neurosurgical assessments, neuropsychological testing, and seizure diary reviews. In this study, responders were defined as exhibiting ≥50% reduction in seizure frequency (PuM‐DBS_resp_) at 1‐year follow‐up compared to a 3‐month baseline period,^9^ whereas nonresponders showed <50% reduction (PuM‐DBS_non‐resp_).

### 2.7 Statistical analysis

Analyses were performed using GraphPad Prism (v10.3.1, Windows) and Jamovi (v2.7.7, Windows). Normality and homogeneity of variance were assessed with the Shapiro–Wilk and Levene tests. Depending on assumption validity, group comparisons were conducted using independent‐samples *t*‐tests or Mann–Whitney *U*‐tests. Statistical significance was set at *p* < .05. To control for multiple comparisons, *p*‐values were adjusted using the Benjamini–Hochberg false discovery rate (FDR) procedure with a significance threshold of *q* < .05.

## 3 RESULTS

### 3.1 Cohort and outcomes

Patients' characteristics are summarized in Table 1. Six patients (four female, two male) were included, as opposed to the SEEG and DBS historical cohorts of the center, which tend to have a sex ratio close to 1:1. Mean age at seizure onset was 18.5 ± 14.5 years (range = 3–47), and at SEEG, 42 ± 12.1 years (range = 26–57). All patients had an intelligence quotient level > 65 and had accomplished at least secondary education, which is in line with the local SEEG and DBS historical cohorts (Details in Supplementary Materials–Explanatory Tables, Table S3 for social–cognitive status). Cerebral MRI showed no macroscopic lesion in four patients, complex cortical malformation (right temporal–parietal–occipital incomplete schizencephaly, polymicrogyria, and periventricular nodular heterotopias) in one patient, and bilateral frontal and temporal–parietal–occipital periventricular nodular heterotopias. The SEEG‐defined EZN was bilateral in three cases. All six included patients had SEEG sampling of the PuM, with eight PuM explored in total: bilaterally in two patients and unilaterally in four patients. SEEG exploration covered both the PuM and the ipsilateral EZN in four patients, whereas in the other two patients the EZN was bilateral, but PuM sampling was unilateral. Mean epilepsy duration prior to DBS implantation was 26.3 ± 13.6 years (range = 13–49). Two patients were PuM‐DBS_resp_ at 1‐year follow‐up. There was a general reduction in seizure frequency regardless of seizure type, including focal seizures with preserved consciousness (with or without observable manifestations), focal seizures with impaired consciousness (with or without falls), and focal to bilateral tonic–clonic seizures. In Patient 1, seizure frequency decreased in parallel with a reduction in seizure severity, with fewer episodes associated with impaired consciousness. Conversely, Patient 5 exhibited an increase in seizures with impaired consciousness (despite a reduction in global seizure frequency), and Patient 3 showed a higher frequency of seizures with preserved consciousness and observable manifestations (despite a reduction in the frequency of seizures with preserved consciousness and without observable manifestations). Among the three patients who experienced focal to bilateral tonic–clonic seizures or focal seizures with impaired consciousness causing a fall, the frequency of such severe seizures decreased in two cases and remained stable in one. None of the patients experienced status epilepticus during baseline or at 1‐year follow‐up. However, given the heterogeneity of seizure types and the limited number of patients, we cannot definitely assert a correlation with the degree of ictal PuM involvement (EZN/PZN/NIZ; detailed in Supplementary Materials–Explanatory Tables, Table S4). Psychological side effects were evaluated using the Neurological Disorders Depression Inventory for Epilepsy and Generalized Anxiety Disorder‐7 scales, available for four of six patients. Changes in these scores were evenly distributed; two patients showed improvement, and two showed worsening. One responder exhibited improvement on both scales at 1 year, whereas among the three nonresponders, two showed worsening and one improvement (details are provided in Supplementary Materials–Explanatory Tables, Table S5). Visual inspection of the stimulating contact locations indicated that contacts in responders were positioned slightly more posteriorly within the PuM compared to those in nonresponders.

**TABLE 1:** Patient clinical, paraclinical, and follow‐up characteristics.

ID	Sex	Age at PuM‐DBS, years	Epilepsy duration until PuM‐DBS, years	Etiology	Epileptogenic zone network	Epilepsy side	Ictal PuM involvement (visual and quantified)	PuM‐DBS status at 12 months
1	F	56	33	Unknown	Insular–parietal	Left	EZN (left PuM)	Resp
2	M	60	13	Unknown	Temporal–insular	Left	PZN (left PuM)	Non‐resp
3	M	38	35	Unknown	Temporal	Bilateral	EZN (right PuM) PZN (left PuM)	Non‐resp
4	F	54	49	Right parietal incomplete schizencephaly and temporal–parietal–occipital polymicrogyria and periventricular nodular heterotopias	Temporal–parietal–insular Normotopic–heterotopic	Left	PZN (left and right PuM)	Non‐resp
5	F	29	14	Bilateral frontal and temporal–parietal–occipital periventricular nodular heterotopias^a^	Temporal–frontal–parietal Normotopic–heterotopic	Bilateral	NIZ (left PuM)	Non‐resp
6	F	32	14	Unknown	Temporal	Bilateral	PZN (right PuM)	Resp

*Note*: Responder = ≥50% reduction in seizure frequency at 1‐year follow‐up compared to a 3‐month baseline period. Non‐responder = <50% reduction in seizure frequency at 1‐year follow‐up compared to a 3‐month baseline period.

Abbreviations: DBS, deep brain stimulation; EZN, epileptogenic zone network; F, female; M, male; NIZ, noninvolved zone network; Non‐resp, nonresponder; PuM, pulvinar medialis thalamic nucleus; PZN, propagation zone network; Resp, responder.

^a^ Patient 5 has filamin A (*FLNA*) mutation.

### 3.2 Interictal and ictal quantifications

**FIGURE 1:**
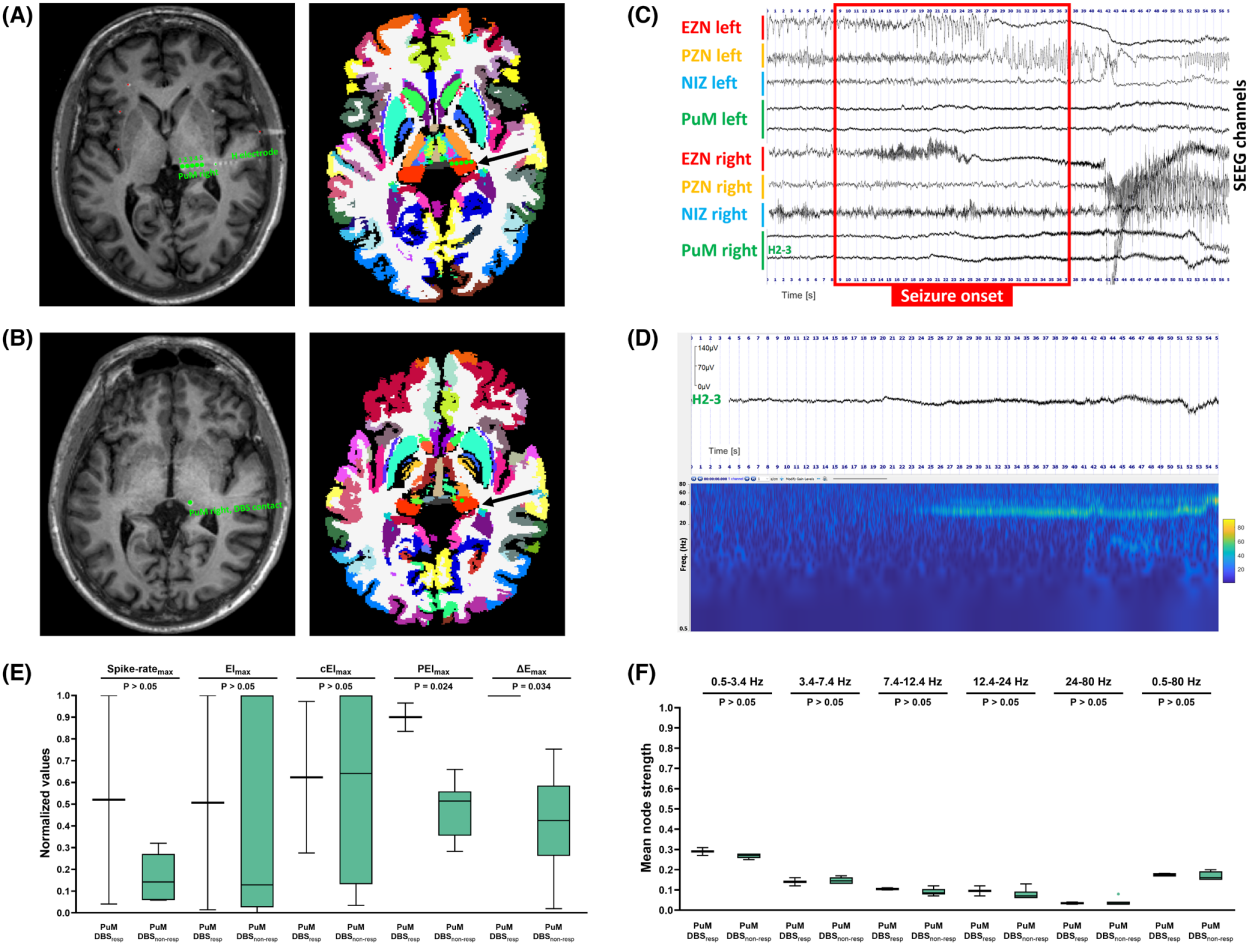
A 35‐year‐old male patient with bilateral temporal nonlesional epilepsy. The patient experienced his first seizure at the age of 3 years, underwent stereoelectroencephalographic (SEEG) exploration at 35 years old, and received pulvinar medialis deep brain stimulation (PuM‐DBS) at 38 years old. Both PuM were sampled during SEEG. The right PuM was classified as part of the epileptogenic zone network (EZN) and the left as part of the propagation zone network (PZN). The patient was a nonresponder to PuM‐DBS at 1‐year follow‐up. (A, left panel) Reconstruction of the H electrode trajectory based on the coregistration of the pre‐SEEG T1‐weighted cerebral magnetic resonance imaging (MRI) and post‐SEEG computed tomography scan. By local standard departmental practice, the H electrode samples the following structures from lateral to mesial: lateral posterior temporal cortex/planum temporale, Heschl's gyrus, and PuM. (A, right panel) Virtual Epileptic Patient atlas including thalamic parcellation projected into the patient's MRI space, with focus on the H electrode contacts sampling PuM. (B) Reconstruction of the right PuM‐DBS lead placement in the patient's MRI space, shown as in panel A. (C) Seizure example illustrating seizure onset and early organization. Each derivation represents a different network: EZN, PZN, or noninvolved zone networks (NIZ). (D) Preictal and ictal visual and time–frequency analysis of one bipolar contact channel pair sampling the PuM. (E, F) Differences in interictal and ictal biomarkers of the pulvinar medialis thalamic nucleus between responders (resp) and nonresponders (non‐resp) to PuM‐DBS. (E) Boxplots displaying the differences between spike rates and epileptogenicity indices (epileptogenicity index [EI], connectivity epileptogenicity index [cEI], permutation entropy index [PEI], delta entropy [ΔE]) between PuM‐DBS_resp_ and between PuM‐DBS_non‐resp_. All values are expressed as their maximum normalized values across all analyzed ictal or interictal segments. (F) Boxplots comparing the mean node strength of PuM, computed as its connectivity with all nodes identified as part of the EZN. Connectivity is computed on the same interictal recordings used for spike rate analysis and is illustrated per frequency band and for the broadband signal.

PuM spike rate_max_ in non‐rapid eye movement sleep, wakefulness, or overall was not statistically significantly different between PuM‐DBS_resp_ and PuM‐DBS_non‐resp_ (Student *t*‐test, FDR‐*p* > .05; Figure 1E). This lack of statistical significance (Student *t*‐test, FDR‐*p* > .05) was also observed when comparing PuM mean node strength between PuM‐DBS_resp_ and PuM‐DBS_non‐resp_ across broadband and subbands, regardless of the sleep–wake status or method used for computation (PuM vs. all other sampled regions, ipsilateral or contralateral regions, or vs. EZN, PZN, or NIZ areas; Figure 1F).

Clinical analysis incorporating EIₘₐₓ, cEIₘₐₓ, and PEIₘₐₓ revealed that PuM was part of the EZN in two patients (one PuM‐DBS_resp_ and the other PuM‐DBS_non‐resp_), PZN in four cases (PuM‐DBS_resp_ and PuM‐DBS_non‐resp_), and NIZ in one patient (PuM‐DBS_non‐resp_). In the two cases with bilateral PuM exploration, both PuMs were PZN in one case, whereas in the other case the right PuM was part of the EZN and the left PuM part of the PZN (Figure 1A–D). PuM EIₘₐₓ and cEIₘₐₓ values did not differ significantly between PuM‐DBS_resp_ and PuM‐DBS_non‐resp_ (Mann–Whitney *U*‐test, FDR‐*p* > .05; Figure 1E). In contrast, both entropy‐based measures were significantly higher in the PuM‐DBS_resp_ group: PuM PEIₘₐₓ (Student *t*‐test, FDR‐*p* = .024, Cohen *d* = 3.35, 95% confidence interval [CI] = .852–5.74) and PuM ΔEₘₐₓ (Student *t*‐test, FDR‐*p* = .034, Cohen *d* = 2.65, 95% CI = .43–4.77; Figure 1E).

## 4 DISCUSSION

We investigated SEEG‐based thalamic biomarkers predictive of response to PuM‐DBS in fDRE. Despite the small cohort (two responders vs. four nonresponders), the heterogeneous epilepsy etiologies, and potential bias related to anatomical variability in electrode placement or recording sites, our findings point to the potential utility of entropy‐based measures.

In curative‐aim epilepsy surgery, SEEG biomarkers—such as thalamic implication at seizure onset, propagation, and epileptogenicity^8^, ^30^—beta‐band connectivity and spike rates^14^ have correlated with seizure prognosis in fDRE. However, such ictal and interictal SEEG features did not differentiate responders from nonresponders in our PuM‐DBS cohort. This contrasts with studies involving interictal and ictal SEEG thalamic connectivity profiles, which did predict responsive neuromodulation outcomes.^31^, ^32^ Moreover, at the individual level, PuM involvement in seizure onset or propagation based on visual SEEG analysis (presence of fast activity at seizure onset as measured by time–frequency maps and/or early changes from background activity identified visually) also failed to reliably predict response, as exemplified by Patient 3 (Table 1, Figure 1C,D). As shown in Table 1, the only patient who exhibited no PuM involvement (low epileptogenicity, NIZ) during seizures was a nonresponder, suggesting that the absence of pulvinar recruitment may be associated with poorer clinical outcomes in PuM‐DBS patients. Conversely, PuM implication ranging from PZN to EZN was present in both responders and nonresponders, suggesting that additional factors are likely to contribute to DBS efficacy, such as the position of the stimulating contacts, which tended to be located more posteriorly in responders in our cohort (although this difference was not formally quantified due to the limited sample size). In addition, in our cohort stimulation was delivered at 145 Hz and electrode trajectories ensured anatomical–functional matching between the epileptogenic cortical regions and the targeted thalamic nucleus—conditions previously associated with favorable outcomes—yet response remained heterogeneous.^32^, ^33^, ^34^ Although classical SEEG metrics related to epileptogenicity, involvement in seizure initiation–early spread, and connectivity did not differentiate responders from nonresponders, the modest cohort size limits the strength of this negative evidence. Subtle associations between thalamic dynamics and clinical response may have gone undetected due to statistical underpowering.

Interestingly, emerging entropy‐based SEEG biomarkers^17^, ^18^ appear promising. Both the ratio of maximum postictal to minimum ictal entropy (PEI) and the difference between baseline mean and minimum ictal entropy (ΔE) were significantly higher in PuM‐DBS responders. Whereas ΔE quantifies the depth of ictal signal simplification—from baseline complexity to the highly organized low‐entropy ictal state—PEI expresses the magnitude of the postictal rebound in entropy relative to this minimum. A larger PEI thus reflects an overshoot of complexity after seizure termination, resembling a stochastic or noiselike signal characteristic of the epileptogenic substrate. Together, these two parameters could describe the bidirectional dynamics of thalamocortical information flow, ΔE indexing the ictal collapse of complexity (pathological synchronization) and PEI indexing the subsequent rebound toward excessive desynchronization. The shared feature of these markers (PEI and ΔE)—the degree of PuM ictal complexity disorganization—may reflect not just involvement, but greater flexibility and responsiveness of corticothalamic networks, potentially facilitating DBS‐induced desynchronization to improve outcomes. This aligns with evidence from acute PuM stimulation during SEEG, which mitigated awareness loss in stimulation‐induced seizures, an effect associated with a smaller ictal SEEG signal complexity decrease^28^ and reduced ictal cortical‐PuM synchronization.^35^

More broadly, our study raises the question of whether SEEG should routinely include thalamic targets. Pulvinar exploration—safe when no additional electrode is required—may be warranted in selected patients, as recent studies have demonstrated the prognostic value of thalamic biomarkers in predicting surgical outcome^14^, ^30^ and potentially DBS response.

## 5 CONCLUSIONS

In our cohort, PEI and ΔE were significantly higher in PuM‐DBS responders, indicating greater ictal PuM engagement in these patients. These findings suggest that identifying the thalamic nucleus with the strongest ictal recruitment—quantified through the two complexity‐based SEEG metrics—may help guide DBS targeting. However, because most current SEEG studies sample only one thalamic nucleus at a time, validation in larger cohorts with multisite thalamic recordings will be essential to determine whether similar patterns hold across nuclei and seizure types. Such datasets could provide the empirical basis for developing virtual or in silico thalamocortical models,^36^, ^37^ enabling mechanistic exploration and optimization of DBS strategies before clinical translation.

## AUTHOR CONTRIBUTIONS

**Ionuț‐Flavius Bratu:** Conceptualization; data curation; formal analysis; investigation; methodology; project administration; software; validation; visualization; writing—original draft; writing—review and editing. **Romain Carron:** Data curation; formal analysis; investigation; methodology; writing—review and editing. **Audrey Clement:** Data curation; formal analysis; investigation; methodology; writing—review and editing. **Samuel Medina Villalon:** Methodology; software; writing—review and editing. **Fabrice Bartolomei:** Conceptualization; data curation; funding acquisition; investigation; methodology; project administration; resources; supervision; validation; writing—original draft; writing—review and editing. **Francesca Pizzo:** Conceptualization; data curation; funding acquisition; investigation; methodology; project administration; resources; supervision; validation; writing—original draft; writing—review and editing.

## FUNDING INFORMATION

The project leading to this publication has received support from the French government under the France 2030 investment plan managed by the French National Research Agency (reference: ANR‐16‐CONV000X/ANR‐17‐EURE‐0029) and from Excellence Initiative of Aix‐Marseille University–A*MIDEX (AMX‐19‐IET‐004).

## CONFLICT OF INTEREST STATEMENT

None of the authors has any conflict of interest to disclose. We confirm that we have read the Journal's position on issues involved in ethical publication and affirm that this report is consistent with those guidelines.

## Supporting information


Data S1.

Data S2.

Data S3.

## Data Availability

GARDEL, AnyWave software, and dedicated plug‐ins (c/EI and Delphos) are available at https://meg.univ‐amu.fr. The VEP atlas is available at https://ins‐amu.fr/vep‐atlas. Data that support our findings are available from the corresponding author upon reasonable request.
