# Supplementary material for: Medial pulvinar stereoelectroencephalographic biomarkers associated with deep brain stimulation response in focal drug‐resistant epilepsy
Source: Epilepsia. 2025 Dec 4;67(1):e8–e17. doi: 10.1111/epi.70046 (PMC12893258; doi:10.1111/epi.70046)
Supplement: Supplementary file 1 — Data S1. [file EPI-67-e8-s003.docx]

**Figure 1.** Distribution of spike-rate values across medial pulvinar (PuM) contacts during the analysed wakefulness and sleep segments.

**Figure 2.** Distribution of Epileptogenicity Index (EI), Connectivity Epileptogenicity Index (cEI), Permutation Entropy Index (PEI), and Delta Entropy (ΔE) values across medial pulvinar (PuM) sampling contacts during the analysed seizures.

**Figure 3.** Position of the pulvinar deep brain stimulation (DBS) lead contacts.


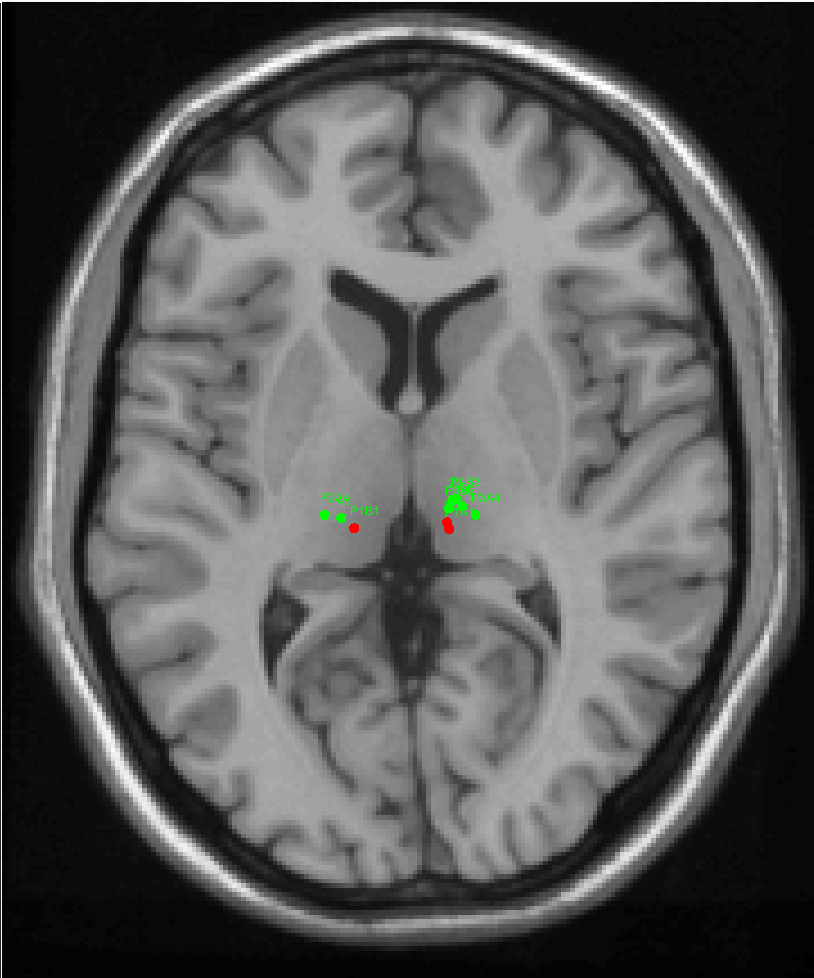

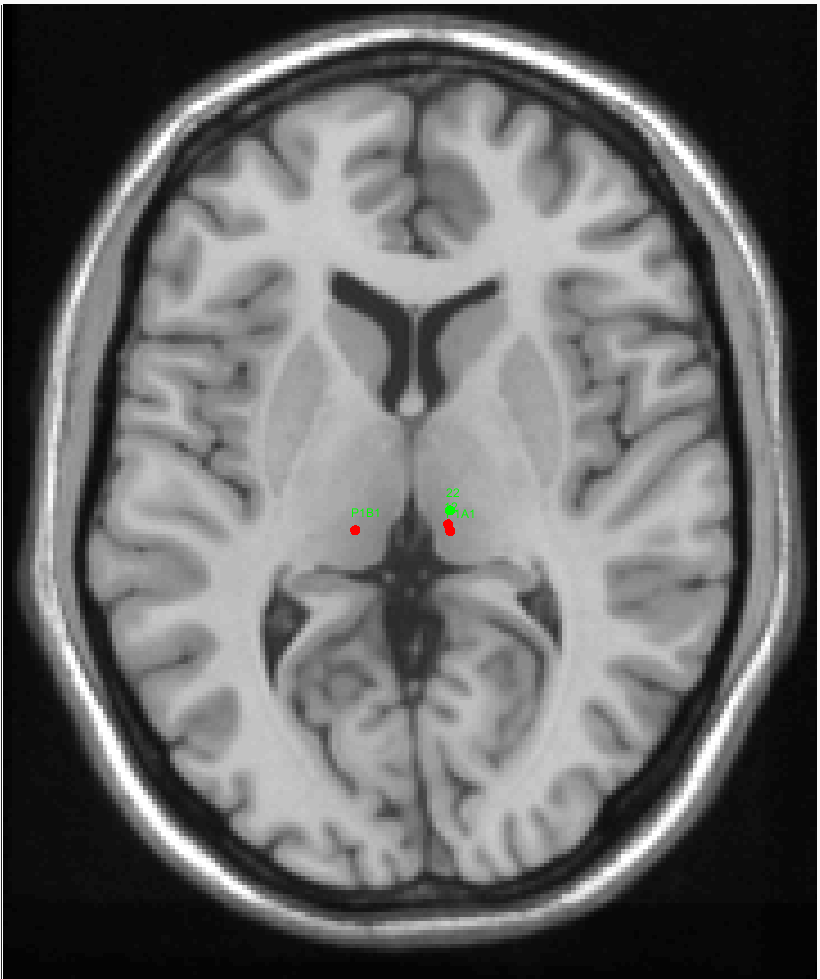

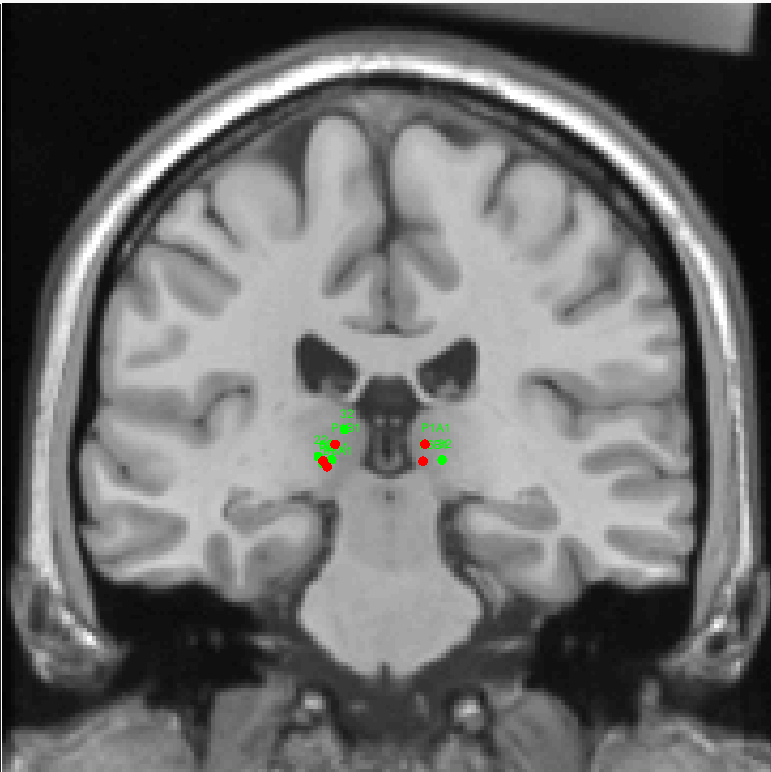

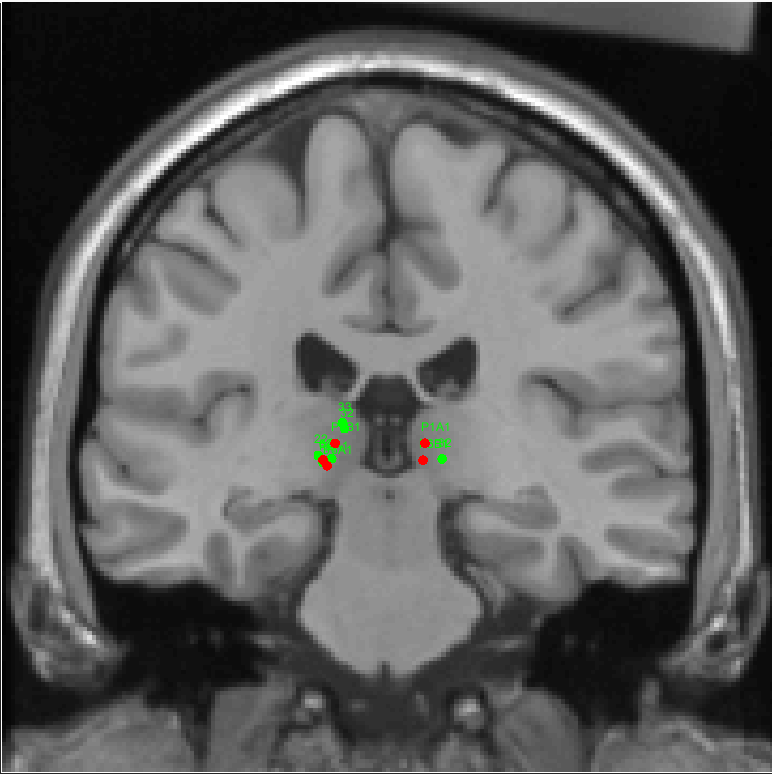

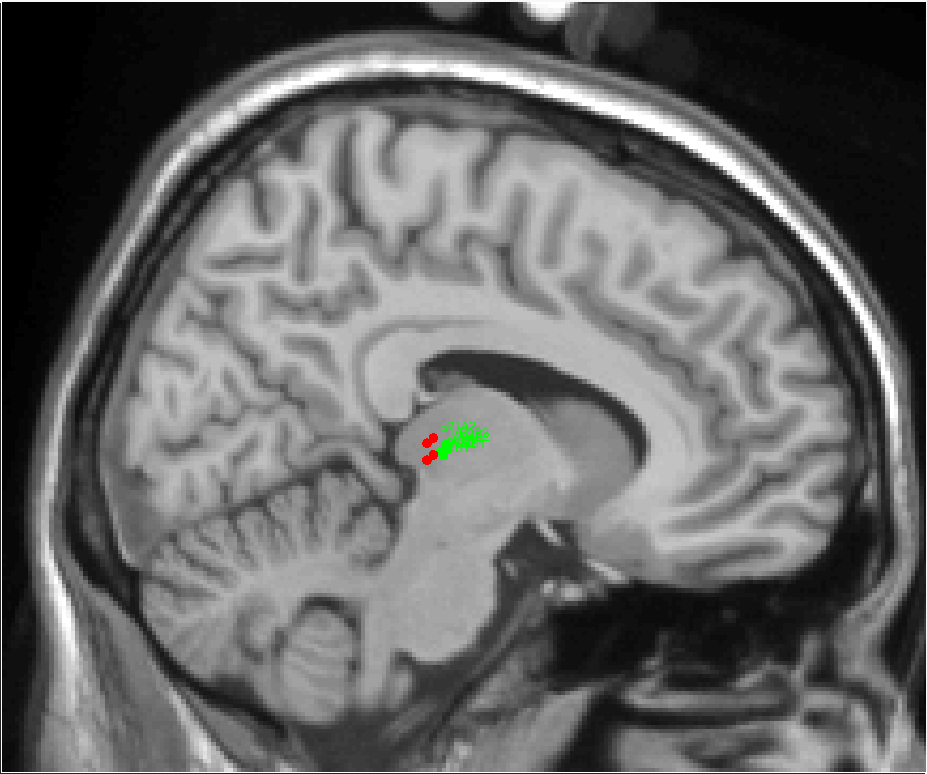

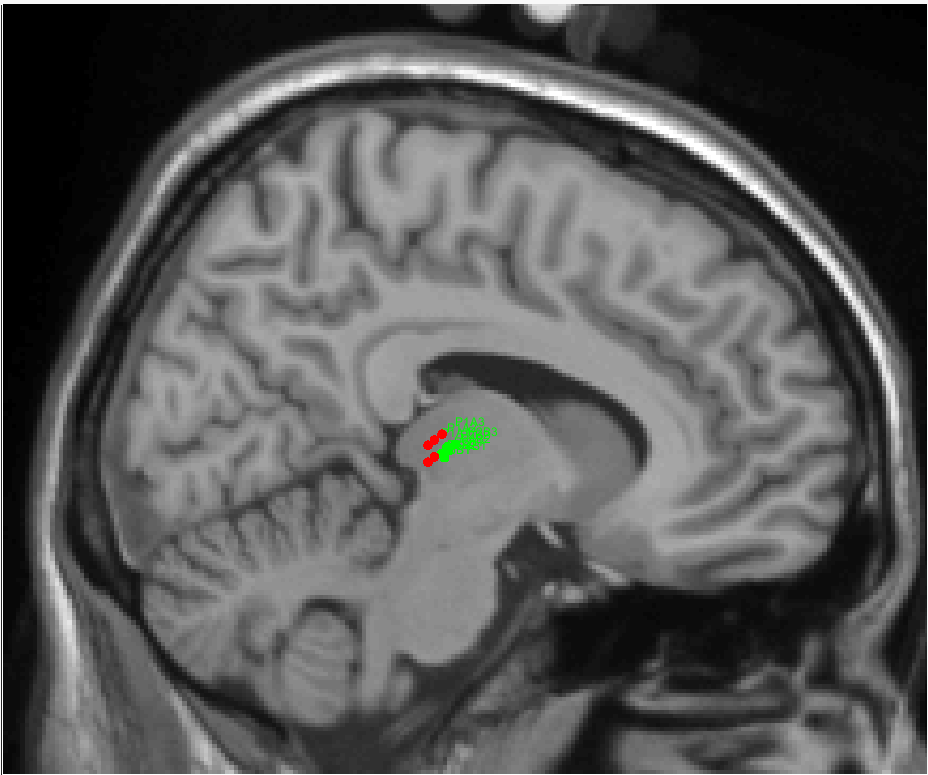


The positions of the DBS leads and their contacts were derived from post-implantation cerebral computed tomography scans. These coordinates were converted into Montreal Neurological Institute (MNI) space and projected onto the MNI Average Brain template (nist.mni.mcgill.ca). **Left column:** positions of all DBS contacts in the cohort. **Right column:** positions of the stimulated contacts in the cohort. **Colour code:** red – DBS responders; green – DBS non-responders.
