# Supplementary material for: Medial pulvinar stereoelectroencephalographic biomarkers associated with deep brain stimulation response in focal drug‐resistant epilepsy
Source: Epilepsia. 2025 Dec 4;67(1):e8–e17. doi: 10.1111/epi.70046 (PMC12893258; doi:10.1111/epi.70046)
Supplement: Supplementary file 2 — Data S2. [file EPI-67-e8-s002.pdf]

Independent Samples T-Test

Independent Samples T-Test

							95% Confidence Interval				95% Confidence Interval	
		Statistic	df	p	Mean difference	SE difference	Lower	Upper		Effect Size	Lower	Upper
**cElmax**	**Student's t**	0.117	6.00	0.911	0.0437	0.373	-0.869	0.956	Cohen's d	0.0957	-1.51	1.69
	**Mann-Whitney U**	6.00		1.000	0.00928		-0.724	0.938	Rank biserial correlation	0.00		

Note. H_a $\mu_{Responder} \neq \mu_{Non\text{-}responder}$

Assumptions

Normality Test (Shapiro-Wilk)

	W	p
**cElmax**	0.808	0.035

Note. A low p-value suggests a violation of the assumption of normality

Homogeneity of Variances Test (Levene's)

	F	df	df2	p
**cElmax**	0.473	1	6	0.517

Note. A low p-value suggests a violation of the assumption of equal variances

Group Descriptives

	Group	N	Mean	Median	SD	SE
**cElmax**	**Responder**	2	0.624	0.624	0.493	0.348
	**Non-responder**	6	0.580	0.641	0.449	0.183

Plots

cElmax

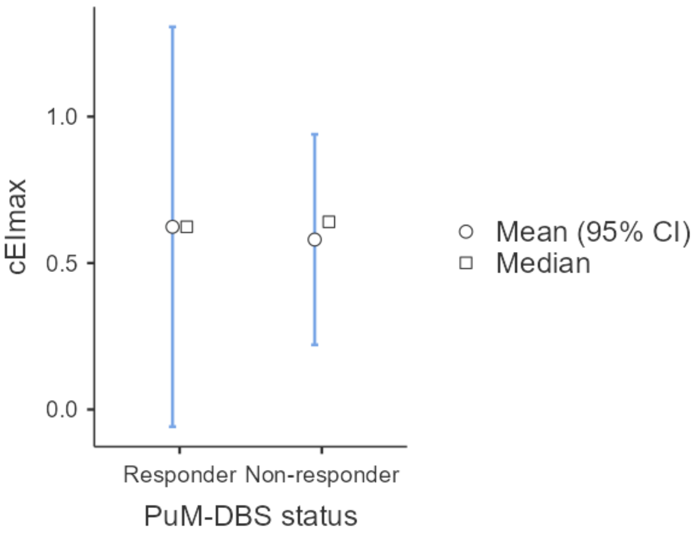
1.0
0.5
0.0
cEImax
Responder
Non-responder
PuM-DBS status
Mean (95% CI)
Median

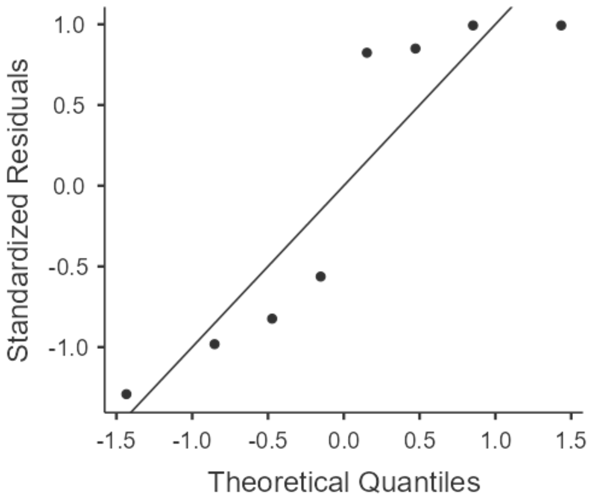
1.0
0.5
0.0
-0.5
-1.0
Standardized Residuals
-1.5
-1.0
-0.5
0.0
0.5
1.0
1.5
Theoretical Quantiles

Independent Samples T-Test

Independent Samples T-Test

		Statistic	df	p	Mean difference	SE difference	95% Confidence Interval			Effect Size	95% Confidence Interval	
							Lower	Upper			Lower	Upper
**Delta-entropymax**	**Student's t**	3.25	6.00	0.017	0.584	0.180	0.144	1.02	Cohen's d	2.65	0.428	4.77
	**Mann-Whitney U**	0.00		0.065	0.584		0.247	0.981	Rank biserial correlation	-1.00		

Note. Hₐ $\mu_{Responder} \neq \mu_{Non\text{-}responder}$

Assumptions

Normality Test (Shapiro-Wilk)

	W	p
**Delta-entropymax**	0.904	0.315

Note. A low p-value suggests a violation of the assumption of normality

Homogeneity of Variances Test (Levene's)

	F	df	df2	p
**Delta-entropymax**	1.81	1	6	0.227

Note. A low p-value suggests a violation of the assumption of equal variances

Group Descriptives

	Group	N	Mean	Median	SD	SE
**Delta-entropymax**	**Responder**	2	1.00	1.00	0.00	0.00
	**Non-responder**	6	0.416	0.424	0.241	0.0985

Plots

Delta-entropymax

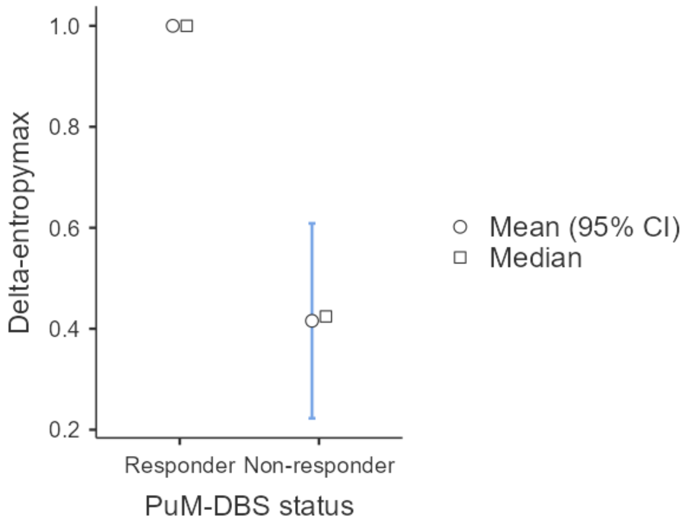

1.0
0.8
0.6
0.4
0.2
Delta-entropymax
Responder
Non-responder
PuM-DBS status
Mean (95% CI)
Median

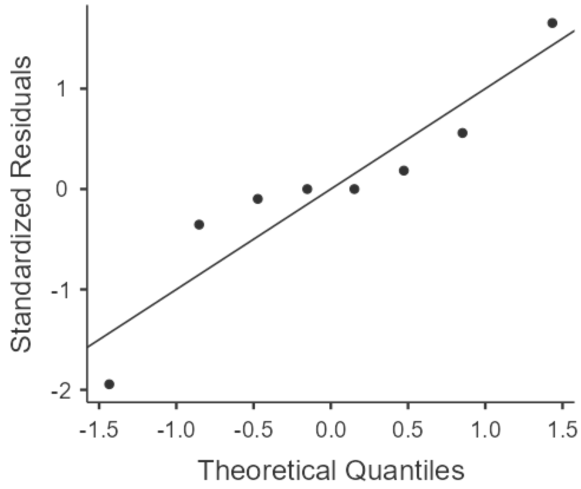

1
0
-1
-2
Standardized Residuals
-1.5
-1.0
-0.5
0.0
0.5
1.0
1.5
Theoretical Quantiles

Independent Samples T-Test

Independent Samples T-Test

		Statistic	df	p	Mean difference	SE difference	95% Confidence Interval			Effect Size	95% Confidence Interval	
							Lower	Upper			Lower	Upper
**EImax**	**Student's t**	0.291	6.00	0.781	0.125	0.429	-0.925	1.17	Cohen's d	0.238	-1.38	1.83
	**Mann-Whitney U**	6.00		1.000	-7.67e−6		-0.986	1.00	Rank biserial correlation	0.00		

Note. Hₐ $\mu_{Responder} \neq \mu_{Non\text{-}responder}$

Assumptions

Normality Test (Shapiro-Wilk)

	W	p
**EImax**	0.790	0.023

Note. A low p-value suggests a violation of the assumption of normality

Homogeneity of Variances Test (Levene's)

	F	df	df2	p
**EImax**	0.393	1	6	0.554

Note. A low p-value suggests a violation of the assumption of equal variances

Group Descriptives

	Group	N	Mean	Median	SD	SE
**EImax**	**Responder**	2	0.507	0.507	0.697	0.493
	**Non-responder**	6	0.382	0.128	0.484	0.197

Plots

EImax

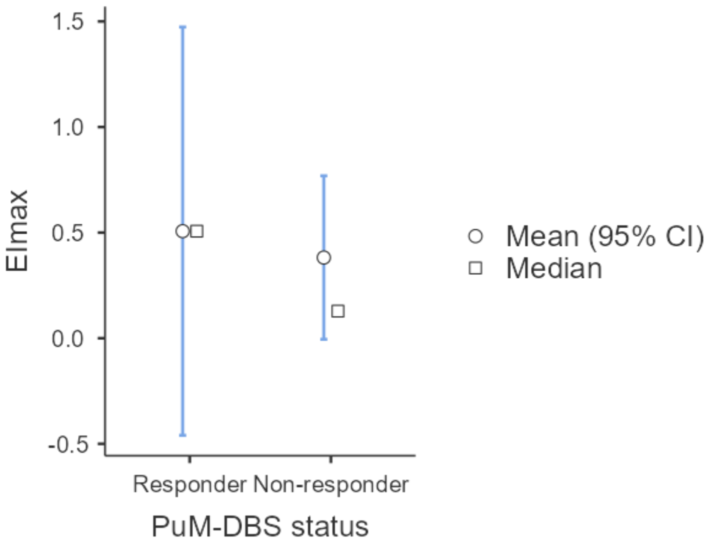
1.5
1.0
0.5
0.0
-0.5
EImax
Mean (95% CI)
Median
Responder
Non-responder
PuM-DBS status

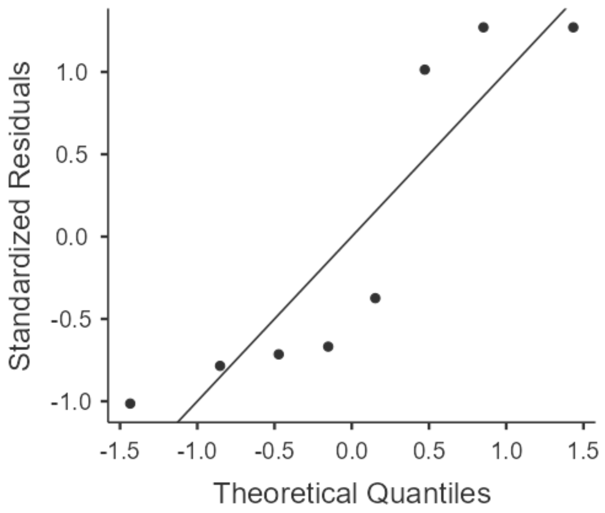
1.0
0.5
0.0
-0.5
-1.0
Standardized Residuals
-1.5
-1.0
-0.5
0.0
0.5
1.0
1.5
Theoretical Quantiles

Results

Independent Samples T-Test

Independent Samples T-Test

		Statistic	df	p	Mean difference	SE difference	95% Confidence Interval			Effect Size	95% Confidence Interval	
							Lower	Upper			Lower	Upper
**PEImax**	**Student's t**	4.10	6.00	0.006	0.420	0.102	0.170	0.671	Cohen's d	3.35	0.852	5.74
	**Mann-Whitney U**	0.00		0.067	0.446		0.175	0.683	Rank biserial correlation	-1.00		

Note. H_a $\mu_{Responder} \neq \mu_{Non\text{-}responder}$

Assumptions

Normality Test (Shapiro-Wilk)

	W	p
**PEImax**	0.956	0.771

Note. A low p-value suggests a violation of the assumption of normality

Homogeneity of Variances Test (Levene's)

	F	df	df2	p
**PEImax**	0.374	1	6	0.563

Note. A low p-value suggests a violation of the assumption of equal variances
[4]

Group Descriptives

	Group	N	Mean	Median	SD	SE
**PEImax**	**Responder**	2	0.900	0.900	0.0925	0.0654
	**Non-responder**	6	0.479	0.515	0.131	0.0535

Plots

PEImax

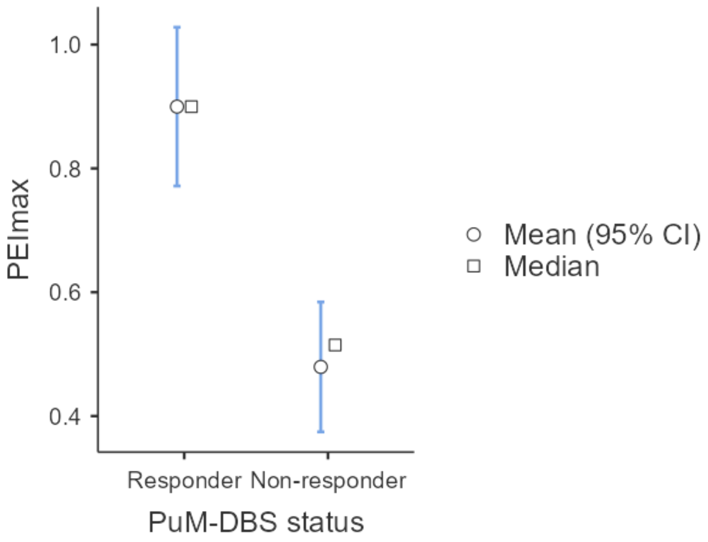

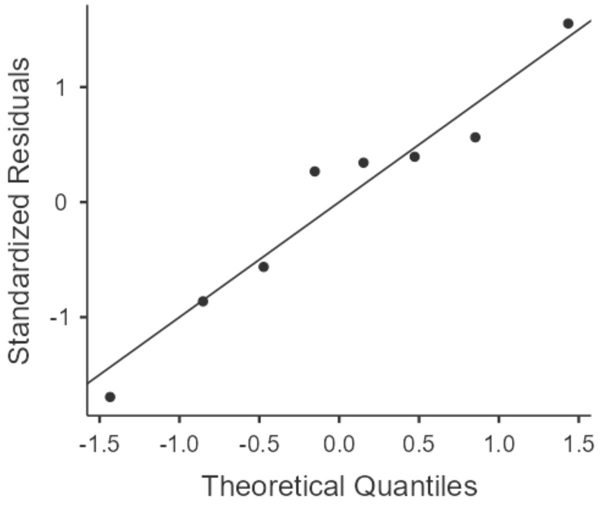

References

[1] The jamovi project (2025). *jamovi*. (Version 2.7) [Computer Software]. Retrieved from https://www.jamovi.org.

[2] R Core Team (2025). *R: A Language and environment for statistical computing*. (Version 4.5) [Computer software]. Retrieved from https://cran.r-project.org. (R packages retrieved from CRAN snapshot 2025-05-25).

[3] Kerby, D. S. (2014). The simple difference formula: An approach to teaching nonparametric correlation. *Comprehensive Psychology, 3*, 2165–2228.

[4] Fox, J., & Weisberg, S. (2024). *car: Companion to Applied Regression*. [R package]. Retrieved from https://cran.r-project.org/package=car.
