# Supplementary material for: Medial pulvinar stereoelectroencephalographic biomarkers associated with deep brain stimulation response in focal drug‐resistant epilepsy
Source: Epilepsia. 2025 Dec 4;67(1):e8–e17. doi: 10.1111/epi.70046 (PMC12893258; doi:10.1111/epi.70046)
Supplement: Supplementary file 3 — Data S3. [file EPI-67-e8-s001.docx]

| **Table 1.** Video-SEEG data. | | | |
| --- | --- | --- | --- |
| **ID** | **Duration of the**  **video-SEEG recording** | **Electro-graphical**  **seizure pattern** | **Number of**  **analysed seizures** |
| P1 | 10 days | One type | 3 |
| P2 | 10 days | One type | 3 |
| P3 | 10 days | One type | 3 |
| P4 | 11 days | One type | 3 |
| P5 | 12 days | Three types | 3  (one of each type) |
| P6 | 11 days | Two types | 3  (out of 3 spontaneous seizures recorded) |

SEEG-stereo-electroencephalography.

| **Table 2.** Pulvinar deep brain stimulation titration side effects. | | | | |
| --- | --- | --- | --- | --- |
| **ID** | **Titration side effect** | **PuM-side** | **Threshold in mA** | **Contact** |
| P1 | None | NA | 4.5 mA (maximum) | NA |
| P2 | None | NA | 2.5 mA (maximum) | NA |
| P3 | Dysesthesia left upper limb | Right | 3.5 | 1 |
|  | Dysesthesia left arm | Right | 1.8 | 2 |
|  | Dysesthesia left leg | Right | 2.0 | 3 |
|  | Paraesthesia left upper limb | Right | 0.7 | 4 |
| P4 | Eye level facial paraesthesia | Right | 2.3 | 1 |
|  | Eye level facial paraesthesia | Right | 2.4 | 2 |
|  | Facial paraesthesia | Right | 2.5 | 3 |
|  | Right hand paraesthesia | Right | 2.6 | 4 |
|  | Right hand and leg paraesthesia | Left | 3.9 | 4 |
| P5 | Left hand paraesthesia | Right | 3.5 | 1 |
|  | Left hand paraesthesia | Right | 2.7 | 2 |
|  | Left hand paraesthesia | Right | 2.9 | 3 |
|  | Left hand paraesthesia | Right | 2.7 | 4 |
|  | Right hand paraesthesia | Left | 3.7 | 1 |
|  | Right hand paraesthesia | Left | 2.2 | 2 |
|  | Right hand paraesthesia | Left | 1.8 | 3 |
|  | Right hand paraesthesia | Left | 1.5 | 4 |
| P6 | Bilateral finger paraesthesia | Right | 1.5 | 1 |
|  | Left upper limb paraesthesia | Right | 1.3 | 2 |
|  | Bilateral hand paraesthesia | Right | 1.4 | 3 |
|  | Left arm paraesthesia | Right | 1.4 | 4 |
|  | Right arm paraesthesia | Left | 3.0 | 3 |
|  | Right arm paraesthesia | Left | 1.2 | 4 |

PuM – pulvinar medials thalamic nucleus; NA – not applicable.

| **Table 3.** Patients’ social-cognitive status at pulvinar deep brain stimulation implantation. | | |
| --- | --- | --- |
| **ID** | **Pre-PuM-DBS**  **Full-scale IQ** | **Education level as per the World Bank classification** |
| P1 | 66 | Lower secondary |
| P2 | 64 | Post-upper secondary vocational qualifications |
| P3 | 89 | Post-upper secondary vocational qualifications |
| P4 | 73 | Post-upper secondary vocational qualifications |
| P5 | 77 | Master’s degree |
| P6 | 76 | Post-upper secondary vocational qualifications |

PuM – pulvinar medials thalamic nucleus.

| **Table 4.** Patient seizure type evolution before and after pulvinar deep brain stimulation. | | | | |
| --- | --- | --- | --- | --- |
| **ID** | **Seizure type** | **Baseline (seizures/month)** | **1-year follow-up**  **(seizures/month)** | **Ictal PuM involvement (visual and quantified)** |
| P1 | A | 0 | 0 | EZN  (left PuM) |
|  | B | 0 | 2 |  |
|  | C | 6 | 3 |  |
|  | D | 27 | 0 |  |
|  | Status epilepticus | 0 | 0 |  |
| P2 | A | 0 | 0 | PZN  (left PuM) |
|  | B | 0 | 0 |  |
|  | C | 4 | 3 |  |
|  | D | 0 | 0 |  |
|  | Status epilepticus | 0 | 0 |  |
| P3 | A | 5 | 3 | EZN  (right PuM)  PZN  (left PuM) |
|  | B | 10 | 33 |  |
|  | C | 0 | 0 |  |
|  | D | 0 | 0 |  |
|  | Status epilepticus | 0 | 0 |  |
| P4 | A | 0 | 0 | PZN  (left and  right PuM) |
|  | B | 0 | 0 |  |
|  | C | 5 | 3 |  |
|  | D | 6 | 4 |  |
|  | Status epilepticus | 0 | 0 |  |
| P5 | A | 0 | 0 | NIZ  (left PuM) |
|  | B | 4 | 0 |  |
|  | C | 0 | 3 |  |
|  | D | 1 | 1 |  |
|  | Status epilepticus | 0 | 0 |  |
| P6 | A | 0 | 0 | PZN  (right PuM) |
|  | B | 0 | 0 |  |
|  | C | 4 | 0 |  |
|  | D | 0 | 0 |  |
|  | Status epilepticus | 0 | 0 |  |

A – focal preserved consciousness seizure, without observable manifestations;

B – focal preserved consciousness seizure, with observable manifestations;

C – focal impaired consciousness seizure, not causing a fall;

D – focal impaired consciousness seizure, causing a fall or focal-to-bilateral tonic-clonic seizure.

PuM – pulvinar medialis thalamic nucleus, EZN – epileptogenic-zone network, PZN – propagation-zone network, NIZ – non-involved-zone network.

| **Table 5.** Pulvinar deep brain stimulation psychological side effects. | | | | | |
| --- | --- | --- | --- | --- | --- |
| **ID** | **Neurological Disorders Depression Inventory for Epilepsy at baseline** | **Neurological Disorders Depression Inventory for Epilepsy at**  **1-year follow-up** | **Generalized Anxiety**  **Disorder-7**  **at baseline** | **Generalized Anxiety Disorder-7**  **at 1-year follow-up** | **PuM-DBS**  **status at**  **at 1-year follow-up** |
| P1 | 22 | 17 | 16 | 16 | Responder |
| P2 | Not available | Not available | Not available | Not available | Non-responder |
| P3 | 15 | 18 | 1 | 8 | Non-responder |
| P4 | 10 | 12 | 3 | 10 | Non-responder |
| P5 | 10 | 7 | 9 | 5 | Non-responder |
| P6 | Not available | Not available | Not available | Not available | Responder |

PuM – pulvinar medialis thalamic nucleus, DBS – deep brain stimulation.
